# Characterization and complete genome sequence of highly lytic phage active against methicillin-resistant *Staphylococcus aureus* (MRSA) isolated from Egypt

**DOI:** 10.1186/s12985-024-02554-0

**Published:** 2024-11-08

**Authors:** Abeer K. Abd El-Tawab, B. A. Othman, A. Sharaf, Samar S. El-Masry, T.F. El-Arabi

**Affiliations:** 1 Department of Agriculture Microbiology, Faculty of Agriculture, Ain Shams University, Cairo, Egypt; 2 Department of Biology, SequAna Core Facility, University of Konstanz, 78464 Konstanz, Germany; 3 Department of Genetics, Faculty of Agriculture, Ain Shams University, Cairo, Egypt

**Keywords:** *Staphylococcus aureus*, MRSA, Antimicrobial resistance (AMR), Phage, Genome sequencing

## Abstract

**Background:**

Methicillin-Resistant *Staphylococcus aureus* (MRSA) is one of the most resistant bacteria to antibiotics. *S. aureus* is an important, widespread pathogen that can cause a variety of infectious diseases in humans and animals. Phages have been recognized as natural, safe, highly specific and effective alternatives agents to antibiotics for preventing and treating bacterial infections caused by MRSA. Therefore, this study aims at the characterization of a novel isolated lytic phage, vB_SauP_ASUmrsa123.

**Methods:**

Isolates of *Staphylococcus aureus* MRSA were obtained on Mannitol Salt Agar and Baird Parker Agar plates and confirmed using VITEK 2. Sewage and clinical samples were used to isolate specific phages for *S. aureus* MRSA, and plaque assays were used for host range determination on Luria-Bertani (LB) media. The phage morphology of the isolated phage was determined by transmission electron microscopy. The phage’s whole genome sequencing was identified.

**Results:**

A total of 25 isolates of Staphylococci were obtained from different clinical sources and showed typical colonies on Baird-Parker and Mannitol Salt Agar plates. The VITEK 2 automated system revealed that all 25 isolates were confirmed as *S. aureus* (MRSA). Two of the most antibiotics-resistant isolates were further confirmed using 16S ribosomal RNA sequencing. A lytic phage was detected against the MRSA isolates tested In Vitro, namely vB_SauP_ASUmrsa123. The phage belonged to *Rountreeviridae* family based on morphological properties observed by TEM and the host range of the isolated phage was tested on the 25 clinical MRSA isolates in Vitro. The one-step growth curve of the isolated phage showed that the latent period was about 55 min, and the burst size was estimated at 167 PFU. The whole genome sequencing and annotation of genes revealed that phage vB_SauP_ASUmrsa123 contained a linear dsDNA with a size of about 17,155 bp with predicted 24 ORFs. Analysis of its genome provides valuable information approximately the variety of phages belonging to the staphylococcal phages class I.

**Conclusion:**

A lytic Podo Phage vB_SauP_ASUmrsa123 was identified against *S. aureus* MRSA isolates and its genome was sequenced. The phage was found to be eligible for potential application in biocontrol.

**Supplementary Information:**

The online version contains supplementary material available at 10.1186/s12985-024-02554-0.

## Introduction

Antibiotic resistance is the urgent topic of the 21st century due to the ever-rising number of hospitalizations resulting from multidrug-resistant (MDR) bacterial infections. A state of emergency has been acknowledged all around the world; the World Health Organization (WHO) cautioned of the possibility of entering an epoch where the antibiotics lose their ability to control the bacterial infections. Antimicrobial resistance (AMR) is the greatest global threat to public health and development. It is estimated that bacterial AMR was directly responsible for 1.27 million global deaths in 2019 [1]. It is predicted that by 2050, there will be no effective antibiotics available, if no new drug is discovered [2].

The most serious and commonly occurring in some of bacteria are *Staphylococcus aureus,* belongs to the family *Micrococcaceae* and is part of the genus *Staphylococcus*, which includes more than 30 species *S. aureus* is by far the most virulent and pathogenic for humans [3]. *Staphylococcus aureus* is a commensal and opportunistic bacterial pathogen that can cause a wide spectrum of infections transferred in healthy individuals [4]. *S. aureus* can infect any site of body, and it is accountable for both minor and severe infections including; skin and soft tissue infections, food poisoning, pneumonia, endocarditis, chronic osteomyelitis, toxic shock syndrome, bacteremia, meningitis and septicemia [5]. Also several local or systemic infectious diseases in animals, such as bovine mastitis, avian arthritis and septicemia [6]. There are still rising number of infectious diseases. Especially, multiple drug-resistant strains of *S. aureus* rise rapidly, such as methicillin-resistant *S. aureus* (MRSA) [7]. MRSA is now a leading cause of nosocomial infections worldwide and has also emerged as a community-associated pathogen [8]. MRSA strains are inherently cross-resistant to virtually all beta-lactam antibiotics, the most effective and widely used class of antimicrobials. According to studies, the species is linked to a broad variety of genes that encode a wide diversity of virulence factors involved in adhesion, host immune evasion, and biofilm formation [9, 10]. Exoenzymes cleave and deactivate immunological components, and toxins causing inflammation and leukocyte death. Each of these elements helps bacteria survive and propagate [11]. Methicillin-Resistant *Staphylococcus aureus* (MRSA) infections are rising in hospitals and the community worldwide, resulting in an increase in medical costs and is resistance to many currently available antibiotics. Therefore, it is necessary to develop effective alternatives to treat MRSA infections. One of the best alternatives that have been used in the treatment of bacterial infection is bacteriophages [12]. Recently, phages have been recognized as natural, safe, very specific and efficient alternatives to antibiotics in preventing and treating bacterial infections caused by *S. aureus*, and they can be used either alone or in combination with other agents like antibiotics (sometimes a combination of phages and antibiotics is better than phage or antibiotics alone). Bacteriophages can used with other antibacterial agents [13, 14]. In human, bacteriophages have been effectively used in the therapy of a wide range of infections in both local and systemic infection [15, 16]. This is due to the fact that they have many features, which give them advantages over antibiotics, e.g., phages multiplying at the site of infection [17]. Phages have the ability to destroy bacterial cells, which makes them a strong antibacterial candidate. Studies including *S. aureus* phage therapy showed effective antimicrobial activity In Vitro and In Vivo [18]. For bacteriophages, the accuracy of annotation has never been more important. Previously, there was no pressing need to know the genome sequencing and an explanation of the phage regions within phage genomes. This was because that antibiotics played a major role in bacterial diseases and mainly relied upon, so interest in phages decreased for a long time, but now the increasing levels of antibiotic resistance in many bacterial pathogens in hospitals. This has led to renewed interest in the exploitation of phages as therapeutic and biocontrol agents So, Sequencing and annotating of proviral regions within bacterial genomes and contributes to our knowledge of the global phage population and the ability to maximize it in the treatment of bacterial diseases. The increasing levels of antibiotic resistance in many bacterial nosocomial pathogens have renewed interest in the exploitation of bacterial viruses as therapeutic [19] and biocontrol agents [20]. In the study of the molecular mechanisms supporting creative infection the sequencing of bacteriophage genomes gives for the description of both close and distant relationships inside the wider population of phages. Since DNA sequencing techniques have progressive over the last the past 20 years, most laboratories can now sequence the genomes of phages [21]. So, the process of sequencing, assembling, and annotating a recently isolated phage is satisfying and adds information to our understanding of the phage population and using them as a good alternative to treating bacterial infections. In this study, a newly isolated lytic phage against MRSA was identified and characterized for the purpose of controlling MRSA infections.

## Materials and methods

### Isolates of *S. aureus* MRSA

Clinical samples were obtained from different clinical sources from suspected MRSA patients that included pus/swab from wounds, sputum in addition to blood were collected from Kasr Al-Ainy Hospital, Cairo, Egypt, according to the standard methods of sample collection (approval ethical code No.11.2024.9) [22]. Blood samples were placed in BACTECTM blood bottles and were incubated at 37 °C in the BACTECTM system for three consecutive days. AzOnly BD BACTEC™ positive samples were sub-cultured on blood agar and incubated at 37 °C for 24 h. While the other sample such as pus/wound swab and urine were inoculated directly into blood agar and incubated at 37 °C for 24h. Suspected *S. aureus* colonies were picked from blood agar plates and transferred onto selective media, Mannitol Salt Agar (MSA) and Baird-Parker Agar (BPA). Typical colonies were further tested by catalase test and coagulase (slide) test.

### Identification of Staphylococcus aureus (MRSA) isolates

Bacterial isolates were further processed for confirmation and identification based on morphological, biochemical, and molecular characteristics. Cefoxtin disc (30 µg) diffusion method [23], and In Vitro antibiotic susceptibility test by Kirby-Bauer disc diffusion method as recommended by CLSI guidelines [24] were carried out. Moreover, VITEK2 automated system was used for bacterial identification and antibiotic susceptibility test [25]. Additional confirmation for bacterial isolates was done by selecting typical colonies grown on Agar (MSA) and Baird-Parker Agar (BPA) plates and analyzed through the MicroSeq^®^ 16S rRNA-based bacterial identification system at Sigma S, Cairo, Egypt. Confirmed isolates were sent to Sigma Scientific Service, Cairo, Egypt for 16S ribosomal RNA sequencing to identify the *Staphylococcus aureus* isolates. Specifically, two isolates from different clinical sources were identified and the Gene Bank nucleotide sequence accession number for partial sequences of 16S rRNA gene was identified with BLAST tool on NCBI (https://blast.ncbi.nlm.nih.gov/Blast.cgi), and they are OQ564500 and OR527118. Additionally, phylogenetic tree was created by Phylogeny.fr [26] and shown in Fig. (S1).

### Isolation of MRSA bacteriophages

Bacteriophages were isolated from raw sewage samples according to previously described method [27]. Briefly,10 mL of exponential phase broth culture of MRSA isolate was mixed with 10 mL of (2×) fresh Luria-Bertani (LB) broth media and 10 mL of centrifuged sewage sample, then incubated overnight at 37 °C with shaking at 100 rpm. The mixtures were incubated at 37 °C for 18 h and centrifuged at 6000 rpm/20 min (Boeco U-320R-Germany) to remove bacteria. The supernatant was filtered through syringe filters 0.22 μm (MCA, CHMLAB, USA) and transferred into sterile tubes. Detection of bacteriophage specific to MRSA and determination of plaque morphology as well as phage titration was done via standard spot test and plaque assay techniques, respectively [28] on solid LB medium at 37 °C.

### Purification of bacteriophage

A single plaque was obtained using sterile cork-borer in 300 µl of SM buffer (5.8 g/L NaCl, 2 g/L MgSO4 7H2O, 50 ml/L of1 M Tris pH 7.5, 5 ml/L of 2% gelatin) and stored at room temperature for overnight to allow the bacteriophage particles to diffuse out of the agar and further titrated on LB plates using plaque assay technique. Biological purification of the phage was carried out by repeating the single-plaque isolation procedure three times until a uniform plaque morphology was achieved on LB agar plates [29]. Purified phages were amplified using liquid propagation on LB broth for 10 h with shaking at 37 °C, then centrifuged at 6000 rpm/15 minutes (Boeco U-320R-Germany). The supernatants were filtered through syringe filters 0.22 μm and finally the phages were stored at 4 °C.

### Determination of MRSA phage host range and efficiency of plating

The host range was determined by spot tests on LB agar plates, and the MRSA isolates were used to determine the phage host range. Ten microliters of the high-titer phage (10^9^ PFU/ml) were spotted on the LB soft agar overlay of the bacteria in the exponential growth phase, then incubated overnight at 37 °C. A clear zone in the bacterial lawn was recorded as complete lysis. To quantify the lytic activity of the MRSA phage, efficiency of plating method (EOP = phage titer on test bacterium/phage titer on host bacterium) was conducted in triplicate on different MRSA isolates as previously described with some modifications. Ten-fold serial dilutions of phage (100 µl) were mixed with 100 µl of the target or host bacterium, then plated as double layers on LB and incubated overnight at 37 °C [30].

### Morphological properties of MRSA phage

The phage particles were loaded onto a carbon-coated copper grid followed by a staining with 2% (w/v) Uranyl acetate (pH 4.9; Sigma, USA). After drying, the grid was observed by high-resolution transmission electron microscope JEOL (JEM 2100, USA) at the Nanotech, Dreamland, Gate 3, 6th October City, Giza, Egypt. Micrographs were taken at an accelerating voltage of 60 KV [31].

### Multiplicity of infection (MOI) assay

Overnight culture of the isolated *S. aureus* MRSA was incubated in fresh LB and incubated at 37 °C with shaking until early exponential growth phase (optical density at 600 nm, 0.4 – 0.6), and mixed with the stock phage at different MOIs ratios of 0.001, 0.01, 0.1, 1.0, and 10. The bacteria was inoculated with the phage with different ratios, and mixtures were kept with shaking (120 rpm) for 5 h at 37 °C. After incubation, the phage samples were centrifuged at 6000 rpm/20 min at 4 °C and the supernatants were filtered through 0.22- µm pore size membranes. The phage titer in the supernatant was determined using a double-layer agar plate method. The optimal MOI was determined when the phage titer reached its highest rate. This assay was performed in triplicate.

### One-step growth curve

Burst sizes and latent periods of the phages were determined by one-step growth experiment according to method described previously, with some modification [32]. The Phage (10^6^ PFU/ml) with *S. aureus* MRSA were incubated at MOI of 0.1 at 37 °C with shaking (150 rpm). Samples were taken at intervals every 5 min for almost 2.5 h. Then, the titration of the mixture was determined every 5 min using the double-layer agar plate method and the data was obtained as following; latent period equal the time post infection until appearing the new progeny particles. The relative burst size was determined according to the equation: Relative burst size = [(Final titre - Initial titre)/ Initial titre] [33]. 

### Phage inhibition assay

The phage was added to the bacterial culture of *S.aureus* MRSA in the exponential growth phase at an MOI of 0.01, 0.1, 1.0 and 10. A 50 µl of *S.aureus* MRSA was added in well of a 96-well plate the bacteria was inoculated with 50 µl phage with different ratios (0.01, 0.1, 1.0 and 10) with negative control (LB broth only) and positive control (bacterial culture of *S.aureus* MRSA). The mixtures were incubated with shaking (100 rpm) at 37 °C for 4 h (Spectro star Nano). The OD_600_ values were measured at a 30-min interval. This assay was performed in triplicate [34].

### Physical properties of MRSA phage

#### Thermal stability

Thermostability of the MRSA phage was tested by the protocol as described [35] with some modifications. Aliqouts of the isolated bacteriophage were incubated at various temperatures ranging from 30 °C to 80 °C. Five ml of phage lysate in test tubes in water bath at 40, 50, 60, 70 and 80 °C for 10 min then directly transferred to ice. The thermal inactivation point (TIP) was further evaluated for MRSA phage by heating the suspensions in water at a narrow range of temperatures of 2-degrees step increase.

### pH Stability

For pH stability assay, phage lysates were mixed in a series of tubes containing SM buffer of different pH values ranging from 3.0 to 11. One ml of phage suspension was added to a 9 ml of SM buffer at different pH levels, and 1N HCl and 1N NaOH were used to adjusted pH values ranging from 3 to 11. and incubated overnight at 37 °C. After incubation, phage lysates were neutralized by1N HCl or 1 N NaOH and phage titer was calculated by double plaque assay according to [36].

### Phage genomic DNA isolation

The phage genomic DNA was extracted utilizing the phenol-chloroform method and subsequently precipitated with ethanol following the protocol outlined by Dácil Rivera and Derek [37, 38]. Initially, a mixture of 1 mL of lysis buffer (composed of 0.5% w/v SDS, 100 µg/mL proteinase K, and 20 mM EDTA) and 10 mL of phage with a concentration of approximately 10^9^ PFU/ml was incubated at 56 °C for one hour. Subsequently, 10 mL of phenol/chloroform isoamyl alcohol was introduced, followed by centrifugation of the solution at 10,000 rpm for ten minutes. The resultant upper aqueous phase was then carefully transferred to a new sterile falcon tube, where an equal volume of isopropanol and 1/10 volume of 3 M sodium acetate were added, and the mixture was left to incubate at -20 °C for 16 h. Following the incubation period, the suspension underwent centrifugation, the supernatant was removed, and the pellet was washed sequentially with 70% ethanol and absolute ethanol before allowing the falcon to air-dry to evaporate residual ethanol. Subsequently, an elution buffer was added to the pellet. The concentration of the extracted DNA was quantified using the FLUOstar Omega microplate reader.

### Phage genome sequencing

Sequencing libraries were prepared according to methods by (Oxford Nanopore Technologies, Oxford, UK), in accordance with the manufacturer’s instructions at Genomics Unit at 57,357 Children Cancer Hospital, Cairo, Egypt. Raw sequencing reads were corrected using NECAT v.0.0.1 (https://github.com/xiaochuanle/NECAT) [39]. The corrected reads were cleaned and trimmed using the Porechop v. 0.2.4 (https://github.com/rrwick/Porechop) [40], which were assembled by the flye Assembler v. 2.9.1 with three polishing iterations (https://github.com/fenderglass/Flye) [41]. The assembly was visualized and validated by Bandage (https://rrwick.github.io/Bandage/) [42]. In addition, LINKS v.1.8.6 (https://github.com/bcgsc/LINKS) [43] was used to scaffold the genome in case of the existence of any internal gaps. Sequence homology analysis and assignment to the phage clusters were performed using BLASTn against the NCBI nucleotide database (https://blast.ncbi.nlm.nih.gov:07/2023) [44]. The genome sequence of vB_SauP_ASUmrsa123 phage was annotated using the PHANOTATE software v. 1.2.1 [45]. Furthermore, protein alignment was used for gene predictions to assure the consistency of the annotation for closely related genomes. For further refinement, frameshifted alignments and partial alignments were processed using a gene prediction program (GeneMarkS+) and final annotations were established by searching against bacteriophage proteins in the SwissProt database [46]. Additionally, the tRNAs were identified using ARAGORN (http://www.ansikte.se/ARAGORN/) [47]. Finally, orthologs profiling of phage vB_SauP_ASUmrsa123 and, three published Staphylococcus phages (GRCS, [NC_023550]; vB_SauR_SW21, [OR683639] and SA44-CTH7, [MK903033]) were identified using OrthoVenn3 [48] utilizing the OrthoFinder algorithm [49]. The single copy orthologs protein sequences were aligned using MUSCLE v5.1 [50] and trimmed using trimAl v1.4 [51]. A maximum-likelihood tree based on the evolution model ‘JTT + CAT’ was inferred using FastTree v2.1.11 [52]. The phage genome was checked for the presence of antibiotic resistance genes (ARG), host virulence genes, and host-genome interaction genes (lysogeny genes) on PhageLeads (https://phageleads.dk/) [53].

## Results

### Isolation and Identification of *Staphylococcus aureus*

Twenty-five bacterial isolates obtained from different clinical sources were confirmed to be gram-positive cocci in grape-similar cluster in gram staining, and characteristic black colonies appeared on Baird-Parker Agar with surrounding clear zone was observed. Also, characteristic colonies appeared conversion of medium from rosy to yellow on Mannitol Salt Agar. In addition, Catalase and Coagulase tests featured positive, which was confirming isolates as *S. aureus*.

### VITEK2 confirmation of Staphylococcus aureus MRSA

A total of 25 *S. aureus* isolates were identified to be MRSA by Cefoxitin antibiotic susceptibility performed by VITEK2 testing system. The results revealed that isolates were *Staphylococcus aureus* MRSA as shown in Table (S1). All *S. aureus* isolates were tested for their sensitivity against more than 15 commonly used antibiotics. Resistance rates of the MRSA isolates were higher towards Benzylpenicillin 25 (100%), Oxacillin 25 (100%). While MRSA isolates showed moderate resistance against each of Tetracycline 14 (56%), Gentamycin14 (56%), Clindamycin 13 (52%), and Erythromycin 13 (52%). The rest of the antibiotics showed less than 50% resistance, such as Ciprofloxacin, levofloxacin 10 (40%). But lower rates of resistance were observed in Trimethoprim/sulfamethoxazole and Rifampicin 7 (28%), 2 (8%). On the other hand, The MRSA isolates showed no resistance against antibiotics Vancomycin, Linezolid, Teicoplanin and Tigecycline (i.e., 100% susceptibility).

### Isolation and morphological properties of phages

**Fig. 1:**
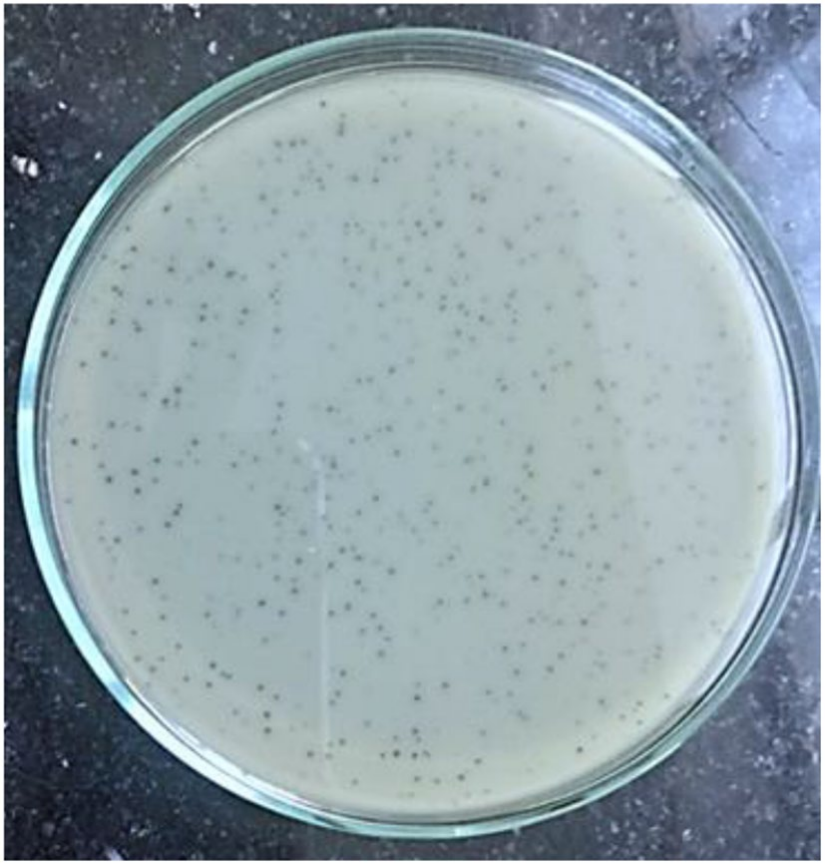
Plaque morphology of phage vB_SauP_ASUmrsa123. Plaque assay plates showed clear plaques of 0.5 – 1 mm in diameter on the specific bacterial host on LB agar media

**Fig. 2:**
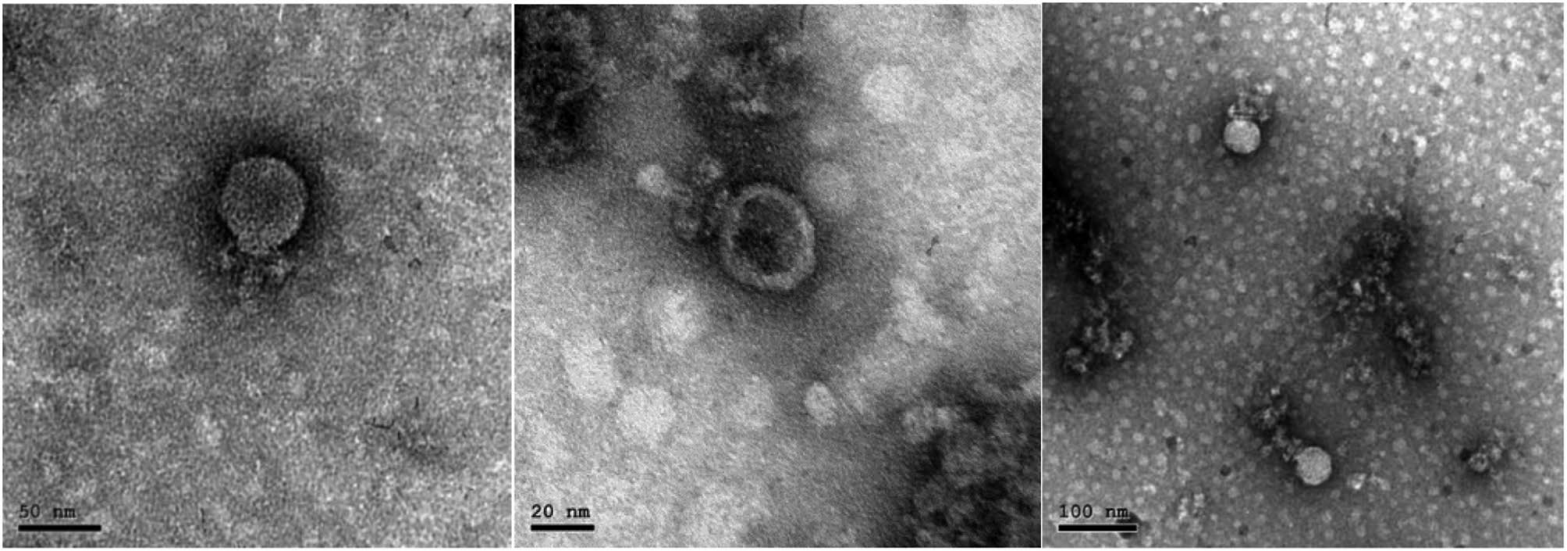
Electron micrographs of phage vB_SauP_ASUmrsa123 showed isometric head and short tail

Plaque assay plates showed clear plaques of 0.5 – 1.0 mm in diameter on the host MRSA as shown in (Fig. 1). The phage particles were examined using high-resolution transmission electron microscopy, and results showed that the isolated phage has binary symmetrical particles with an isometric head of about 55.56 nm and a short tail measuring of about 14.49 nm (Fig. 2).  The isolated phage believed to belonging to family *Rountreeviridae* and was given the name of vB_SauP_ASUmrsa123 (further indicated as ASUmrsa123).

### Host specificity of phage vB_SauP_ASUmrsa123

The efficiency of plating (EOP) was estimated for all MRSA isolates to Phage vB_SauP_ASUmrsa123. The phage showed activity against 12 of the 25 *S.aureus* MRSA isolates. The efficiency of plating (EOP) of phage was grouped into four classes EOP > 0.5 for high production, and medium production as 0.1 < EOP < 0.5, low production as 0.001 < EOP < 0.1, but very low production as EOP < 0.001. High EOP production means that infection of the target bacterium produces at least 50% of the PFU in comparison to the primary host (Table S1).

### Optimal multiplicity of infection (MOI)

MOI was tested with values of 0,001,0.01,0.1,1.0 and 10 The results showed that the optimal MOI of phage VB_SauP_ASUmrsa123 required for the best propagation was 10, which was the highest titer reached by the phage lysates to 4.5 × 10^10^. the results recorded in Table 1.

**Table 1:** Optimal multiplicity of infection (MOI) of phage VB_SauP_ASUmrsa123

CFU of MRSA OQ564500 (main host)	PFU of phage	MOI	titers (PFU/mL) (Mean)
10^8^	10^5^	0.001	4 × 10^7^
10^8^	10^6^	0.01	6 × 10^8^
10^8^	10^7^	0.1	2 × 10^9^
10^8^	10^8^	1.0	2 × 10^10^
10^8^	10^9^	10	4.5 × 10^10^

* Titers (PFU/mLrepresent the mean of three independent experiments for each MOI

### One-step growth curve

**Fig. 3:**
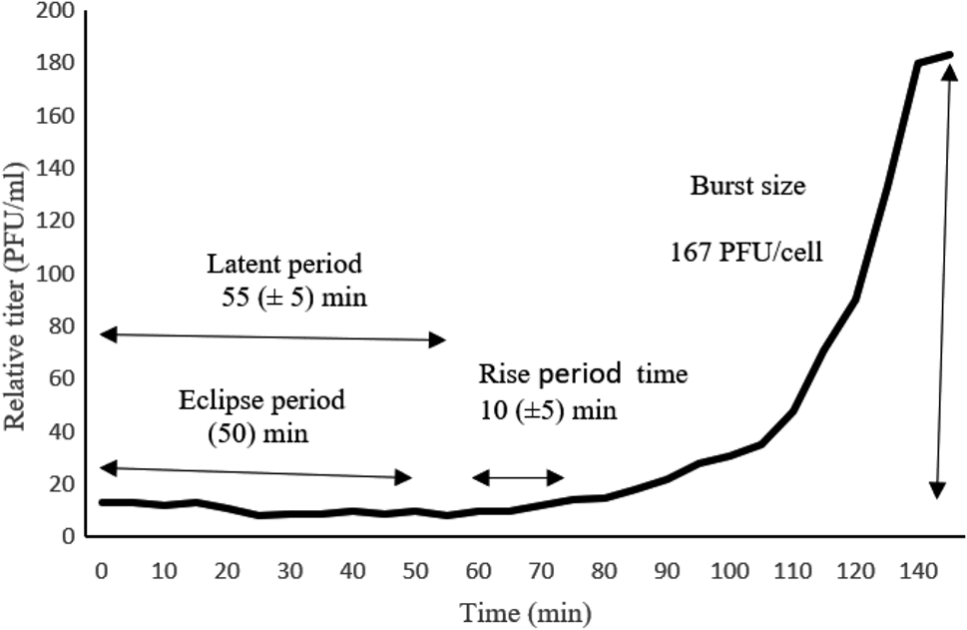
One-step growth curve of vB_SauP_ASUmrsa123 at MOI = 0.1

To evaluate the latent period and burst size of the phage ASUmrsa123, one-step growth experiment was carried out at MOI = 0.1, and the latent period was estimated to be of about 55 min with a burst size of about 167 PFU per infected cell. Eclipse period and rise period time were 50 and 10 min, respectively (Fig. 3).

### Phage inhibition assay

**Fig. 4:**
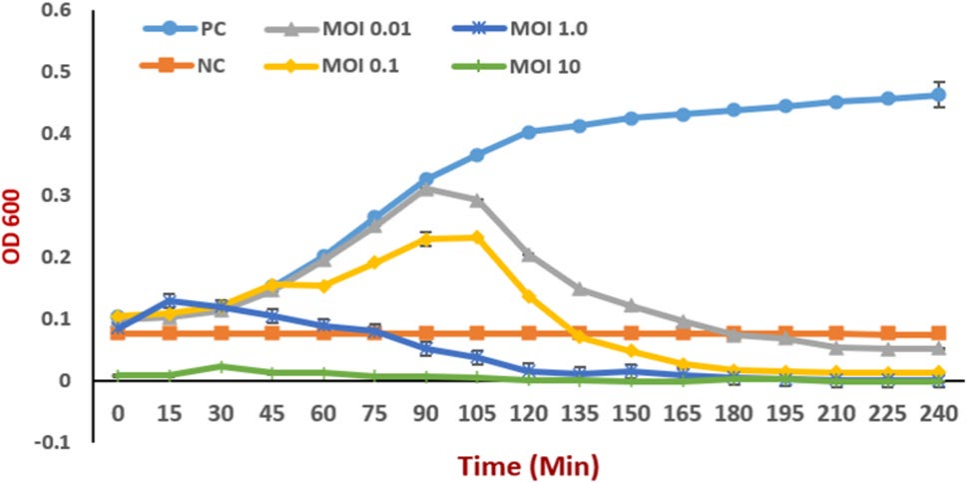
Effect of phage ASUmrsa123 on MRSA OQ546500 growth In Vitro at different MOI’s (0.01, 0.1, 1.0 and 10). MRSA OQ564500 was used as positive control and culture medium without bacteria as a negative control. Data were obtained from three independent experiments and are shown as mean ± SEM. PC: Positive control (*S. aureus* OQ546500,) NC: Negative control (culture medium without bacteria) MOI: Multiplicity of Infection (Treated with phage)

The inhibitory potential of phage ASUmrsa123 on the MRSA host was tested in liquid media. Phage could effectively inhibit the growth of *S. aureus* MRSA OQ564500 after 100 min when the MOI was 0.01; the bacterial growth started declining and reached complete inhibition after 150 min. While at MOI of 0.1, the bacterial growth declined after 120 min and completely stopped after 140 min. Moreover, at MOI 1.0, the bacterial growth remained in the lag phase for about 50 min and then the growth was stopped completely. With MOI 10, the bacteria did not show any sign of growth during the incubation period (Fig. 4).

### Stability of *Staphylococcus aureus* MRSA phage

**Fig. 5:**
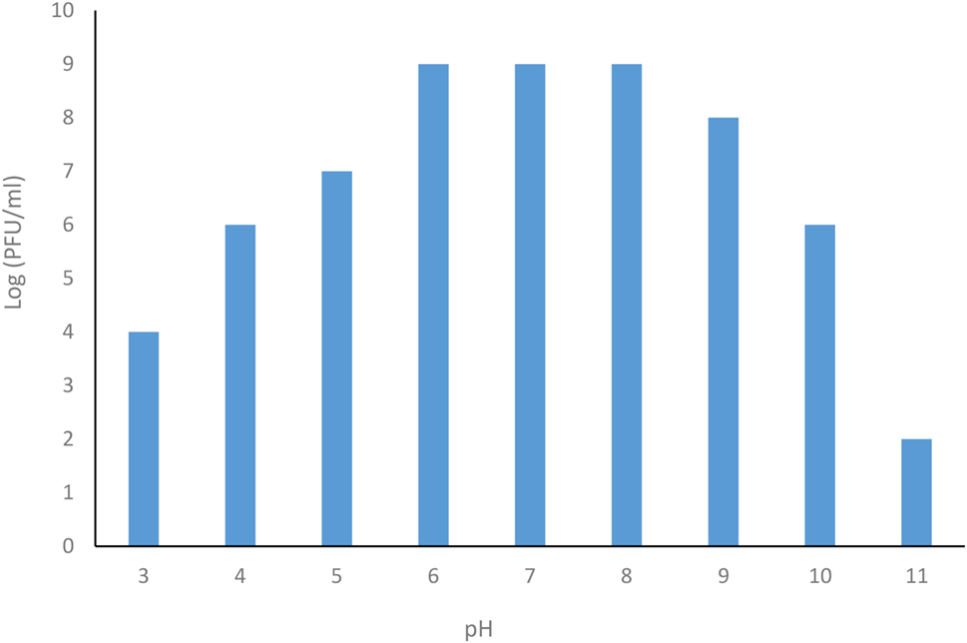
pH stability assay of phage

The thermal stability of the phage vB_SauP_ASUmrsa123 was studied at a wide range of temperatures from 40 °C to 80 °C with intervals 10 °C for 10 min. After incubation the activity of phage was tested, and the results showed that the phage was able to completely inhibit bacterial growth at 74 °C. The phage was stable up to 70 °C for 10 min. The activity decreased gradually, and complete inactivation occurred at 80 °C. Thermal inactivation point (TIP) was determined at 76 °C ± 2. On the other hand, the pH stability was studied in SM buffer. The phage vB_SauP_ASUmrsa123 was evaluated over a wide range of pH level, from 3 to11 for overnight at 37 °C. After incubation, the phage was able to completely inhibit bacterial growth in pH levels ranging from 5.0 to 9.0. Lytic activity was stable between of pH 5 and 10 and a marked decrease was observed at pH 4 or below and beyond pH 10 (Fig. 5).

### Genome analysis of phage vB_SauP_ASUmrsa123

**Fig. 6:**
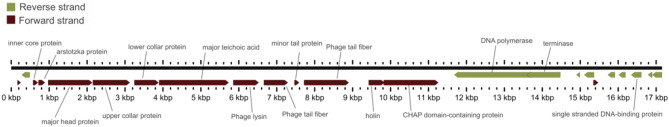
The genome structure of phage vB_SauP_ASUmrsa12. The phage contained of total 24 functional genes, most of which appeared on the forward strand. The phage genome contained 15 predicted functional genes and 9 genes were identified as hypothetical proteins

Genome sequencing data resulted in the presence of one contig, which demonstrated a length of 17,155 bp linear, double-stranded DNA. According to our TEM result, the phage belonged to the *Rountreeviridae* family, which is also confirmed by the genome annotation and using the blast tool against similarly phages in the GenBank database. The phage ASUmrsa123 genome contained 24 Open Reading Frames (ORFs), and their predicted functions according to BLASTx tool on (https://blast.ncbi.nlm.nih.gov/Blast.cgi) showed 15 functional proteins, and 9 hypothetical proteins (Fig. 6).

**Accession number.** The genome sequence of *S. aureus* MRSA phage vB_SauP_ASUmrsa123 was put in GenBank under the accession number OR259390.

The phage genome showed predicted genes that suggested the lytic activity with no predicted genes for lysogeny.

**Fig. 7:**
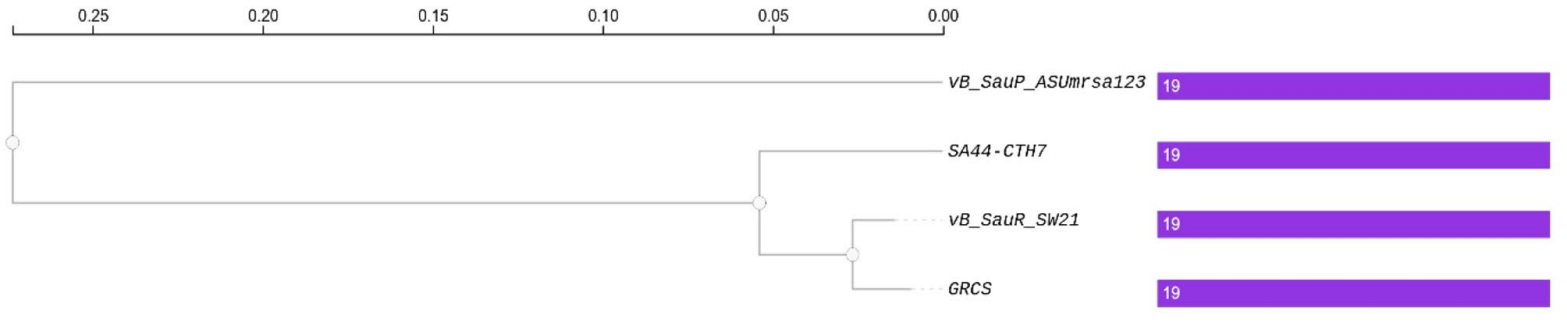
Phylogenetic relationship of vB_SauP_ASUmrsa123 phage and another three highly similar phages SA44-CTH7, GRCS and vB_SauR_SW21. The graph illustrates that despite the high similarity of 19 gene orthologs clusters, the phage vB_SauP_ASUmrsa123 appeared at a different node in the tree, due to distinct presence of additional 5 gene orthologs

**Fig. 8:**
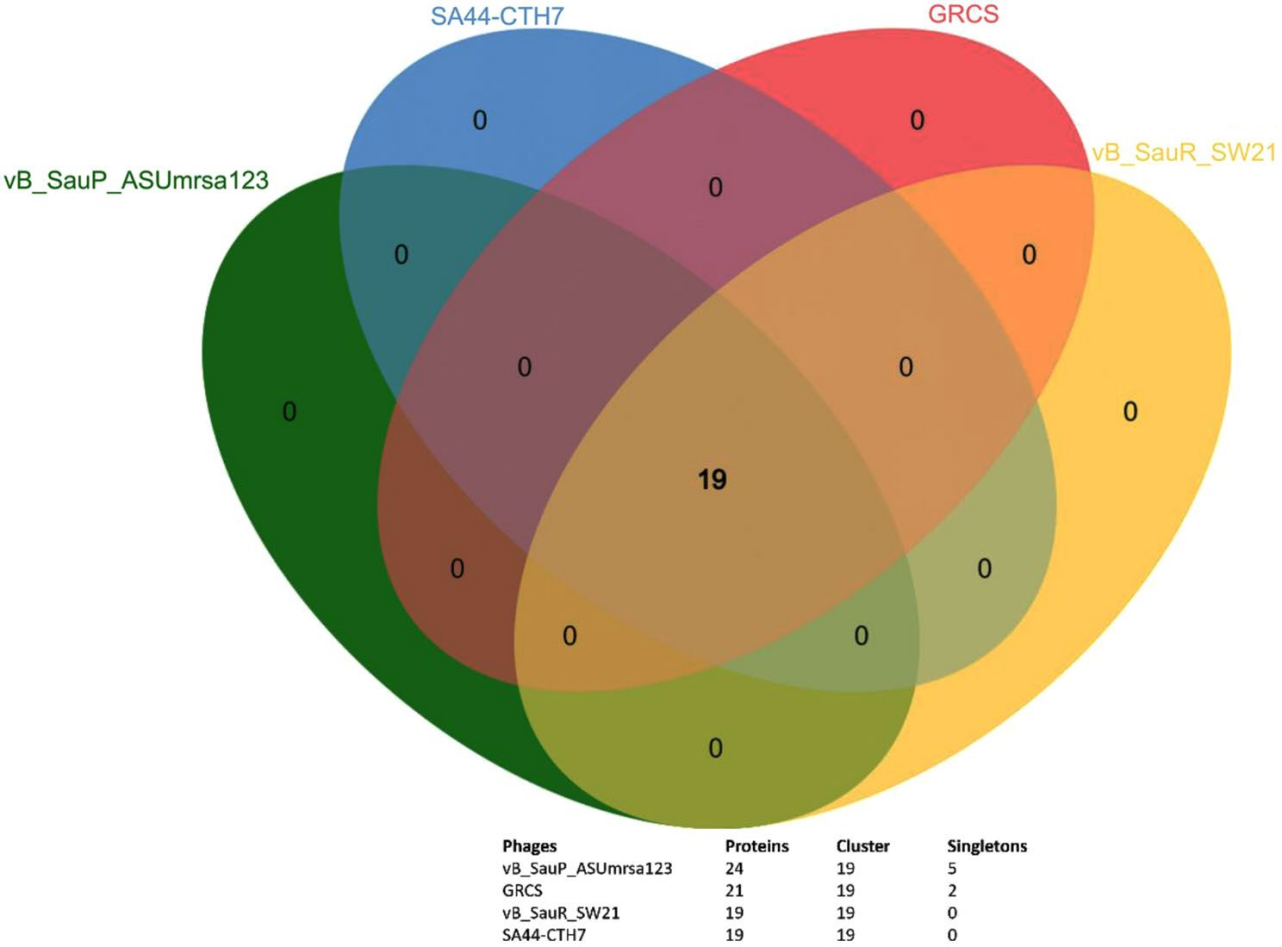
Gene orthologs clusters across *S. aureus* vB_SauP_ASUmrsa123 phage and three highly similar phages SA44-CTH7, GRCS and vB_SauR_SW21. The graph illustrates the location which the four phages have similar 19 orthologs, while phage vB_SauP_ASUmrsa123 has 5 distinct singletons from the other three phages

According to Blastn and Blastx tools, the suggested linage of phage ASUmrsa123 is Viruses; Caudoviricetes; Rakietenvirinae; Rosenblumvirus. Three phages SA44-CTH7, GRCS and vB_SauR_SW21 were identified as highly similar to phage vB_SauP_ASUmrsa123 and their phylogenetic relationship is shown in Fig. 7. Gene orthologs of the four phages showed that the four phages share 19 protein clusters. Nevertheless, the phage ASUmrsa123 has 5 singletones, compared to phage GRCS, which had total 21 protein clusters, with shared 19 and 2 signletones protein clusters. While phage SA44-CTH7 and vB_SauR_SW21 shared all 19 protein clusters identified in their genomes (Fig. 8).

Despite the similarity of the four phages, the phage ASUmrsa123 was the only among the four phages to have predicted gene of the arstotzka capsid protein.

In order to determine the potential for the utilization of phage ASUmrsa123 for therapy or biocontrol purposes, it is crucial to confirm the safety of the phage. The phage genome was checked on PhageLeads for genes related to antibiotic resistance and/or virulence genes and the results showed that there was no evidence of the phage to carry any of these genes as well as any lysogeny genes.

## Discussion

*Staphylococcus aureus* is one of the common pathogens obtained from hospitals and community acquired pathogens, and the reason of the majority of infections, causing superficial skin and soft tissue infections to serious systemic infection leading to illness and death of a person [54]. MRSA is now a leading cause of nosocomial infections worldwide and has also arisen as a community-associated pathogen [8]. MRSA is a major group of multi drug-resistant organisms, which are responsible for rising the rate of morbidity and mortality [55]. This problem is further compounded via development of methicillin resistance.

This study involved the isolation of 25 *S. aureus* MRSA isolates obtained from clinical specimens, which majorly from wound/pus swabs and some from blood and sputum swabs. This has been previously reported where *S. aureus* MRSA might be dominant in wound/pus infections [56]. Results of antimicrobial susceptibility test of S. aureus MRSA isolates shown that all isolate were resistance to Methicillin, by the use of Cefoxitin disc test. Numerous investigators have reported that the results of Cefoxitin disk diffusion tests correlate better with the presence of mecA than do the results of disk diffusion tests using Oxacillin [57]. In addition, the antibiotic resistance profile for all MRSA isolates were 100% susceptible to Vancomycin, Linezolid and Teicoplanin, but all isolates were resistant to β-Lactams (Benzylpenicillin and Qxacillin). While, MRSA isolates showed moderate resistance against each of Gentamycin1, Clindamycin and Erythromycin, which was similar to some studies reported previously [58]. Suitable performance for different types of direct culture approaches in combination with Vitek 2 have been reported in terms of rapid identification of bacteria, reduced antibiotic use [59, 60]. In this study, the partial genome sequence of 16S rRNA gene for two isolates were identified and could be accessible with GenBank accession numbers of OQ564500 and OR527118.

Due to the reappearance of antimicrobial resistance in MRSA, these infections have become riskier and costly in the last 20 years because of the wide use of antibiotics [61]. This rising threat has recently caused interest in the development of phage therapy. One of the advantages of the use of bacteriophages as antibacterial therapeutics is the fact that phages have high specificity. In this study, the main objective was to isolate strongly lytic phage against MRSA. A novel phage vB_SauP_ASUmrsa123 was isolated and identified to have particles had an isometric head of 55.56 nm and short tail measuring 14.49 nm showed morphology like vB_SauP_436A1, CSA13 and SLPW phages [62, 63, 18], which belonged to the family *Rountreeviridae*, and were mainly virulent phages [64]. This may support why the presence of *Rountreeviridae* phages in the commercial therapeutic phage products, which has been also shown in several studies [65].

The host range of the isolated lytic phage was studied qualitatively using spot test; among the 25 clinical MRSA isolates tested, 12 MRSA isolates were lysed by the phage, In Vitro. In addition, the efficiency of plating (EOP) was assessed for all *S. aureus* MRSA isolates to phage vB_SauP_ASUmrsa123. The higher EOP values of the vB_SauP_ASUmrsa123 phage was against 6 isolates EOP > 0.5, one isolate was moderate and five isolates were low production. While, two isolates were very low production and eleven isolates were no production. The differences in EOP of vB_SauP_ASUmrsa123 against diverse tested strains might be because of variations in availability of entry receptors and resistance mechanisms [66]. This results were that to the study reported on TSP phage [67]. Phage ASUmrsa123 had relatively broad inhibition spectrum against MRSA isolates. To quantify the lytic activity of the phage, the efficiency of plating results showed higher EOP values of the vB_SauP_ASUmrsa123 phage against 6 MRSA isolates (EOP > 0.5), one isolate was moderate and five isolates were low production. While, two isolates were very low production and eleven isolates were no production. Phage ASUmrsa123 had relatively broad inhibition spectrum against MRSA isolates, which suggested the potential of the phage to be exploited in combating MRSA infections.

The eclipse and latent periods of vB_SauP_ASUmrsa123 was detected at 50 min and 55 min (± 5), respectively, and the burst size is 167 PFU/ml per infected cell in one-step growth experiments. The results showed that vB_SauP_ASUmrsa123 had a long latent period, which is similar to one reported for vB_SauP-436A1, but the burst size was different [62].

The inhibitory potential of phage ASUmrsa123 on the MRSA host was tested in liquid media. Phage could effectively inhibit the growth of *S. aureus* MRSA OQ564500 by infecting the bacteria at exponential phase for 240 min at different MOIs. The ASUmrsa123 phage reduced bacterial growth in a MOI-dependent manner, as seen by the curves, wherein the higher MOIs exhibited the biggest decrease value. These results are consistent with the previous phages,  such as vB_SauP-436A1 and SPLW [62, 18].

According to the results of various studies, phage’s ability to survive at relatively high temperatures and strong acidic and alkaline environmental conditions are unique features of bacteriophages as biological controllers to bacteria. Also, the application of phages in therapy requires knowledge of the stability of the phages at different temperatures and pH. Moreover, the ability of phages to infect and replicate inside their hosts also depends on temperature [68]. Therefore, thermal stability of the isolated lytic phage for MRSA was studied by exposure of phage suspensions to different temperatures for 10 min. Results showed that phage vB_SauP_ASUmrsa123 had Thermal Inactivation Point (TIP) at 76 °C ± 2. The phage was found to be heat resistant and able to survive in a treatment of 70 °C for 10 min. Long-term storage stability showed that phage vB_SauP_ASUmrsa123 was more viable at 4 °C. Also, phage vB_SauP_ASUmrsa123 was tested for its pH stability, and the phage was found to be stable from pH 4.0 to 10. While, the phage maintained more active over a wide pH range (5 to 9), but under extreme pH conditions (below 5 and above 10), a marked decrease in phage titer was observed. Such results aligned with the results reported in a previous studies with the phages TSP, SLPW and vB_SauP-436A1 [67, 18, 62].

The genome of phage ASUmrsa123 was identified to be linear, double-stranded DNA with a size of 17,155b. Many *S. aureus* phages were reported to have similar type and size of DNA, such as SA44-CTH7, GRCS, vB_SauR_SW21 [69]. The phage ASUmrsa123 genomic characterization identified 24 ORFs involved in packaging, head, tail, lysis, and DNA replication, and it showed high homology with other phages, such as SA44-CTH7, GRCS, vB_SauR_SW21. Based on genome characterization, the suggested linage of phage ASUmrsa123 is: Viruses; Caudoviricetes; Rakietenvirinae; Rosenblumvirus. The phage capsid contained a unique capsid protein arstotzka, which was found in other *S. aureus* phages, such as P68 [70]. As confirmed by PhageLeads, no antibiotic resistance genes, virulence genes or host-genome interaction genes were detected [53]. Similarly, the phage vB_SauS_JS02 was also demonstrated as a potential biocontrol agent against biofilm-producing *S. aureus,* and it may be applicable for phage therapy [71]. This led to a belief that the phage vB_SauP_ASUmrsa123 is strictly lytic. The combined data of phage stability, relatively broad range of infectivity against host isolates, and defined safety based on genome analysis, led to a believe that the phage vB_SauP_ASUmrsa123 is a strong candidate for biocontrol applications. Further research on animal model and In Vivo studies are required to confirm the potential application of this phage in therapeutic purposes.

## Conclusion

In this study, a highly virulent phage specialized against MRSA, and its morphological, biological, and molecular properties were studied. The findings suggested that phage vB_SauP_ASUmrsa123 is a potential candidate for more In Vivo studies to confirm its safety and efficiency in phage therapy of *S. aureus* MRSA infections.

## Electronic supplementary material

Below is the link to the electronic supplementary material.

Supplementary Material 1

## Data Availability

No datasets were generated or analysed during the current study.
