# Supplementary material for: Characterization and complete genome sequence of highly lytic phage active against methicillin-resistant Staphylococcus aureus (MRSA) isolated from Egypt
Source: Virol J. 2024 Nov 8;21:284. doi: 10.1186/s12985-024-02554-0 (PMC11545979; doi:10.1186/s12985-024-02554-0)
Supplement: Supplementary file 1 — Supplementary Material 1 [file 12985_2024_2554_MOESM1_ESM.docx]

**Table S1. Antibiotic susceptibility pattern of methicillin resistant *Staphylococcus aureus* (MRSA) isolates from different clinical samples.**

| *Staphylococcus* isolates | 1 | 2 | 3 | 4 | 5 | 6 | 7 | 8 | 9 | 10 | 11 | 12 | 13 | 14 | 15 | 16 | 17 | 18 | 19 | 20 | 21 | 22 | 23 | 24 | 25 |
| --- | --- | --- | --- | --- | --- | --- | --- | --- | --- | --- | --- | --- | --- | --- | --- | --- | --- | --- | --- | --- | --- | --- | --- | --- | --- |
| Benzylpenicillin | R | R | R | R | R | R | R | R | R | R | R | R | R | R | R | R | R | R | R | R | R | R | R | R | R |
| Cefoxitin | R | R | R | R | R | R | R | R | R | R | R | R | R | R | R | R | R | R | R | R | R | R | R | R | R |
| Ampicillin | R | R | R | R | R | R | R | R | R | R | R | R | R | R | R | R | R | R | R | R | R | R | R | R | R |
| Amikacin | S | R | S | S | R | S | R | R | R | S | S | S | S | R | S | S | R | S | S | R | R | R | R | S | S |
| Vancomycin | S | S | S | S | S | S | S | S | S | S | S | S | S | S | S | S | S | S | S | S | S | S | S | S | S |
| Ciprofloxacin | S | R | S | S | R | S | S | R | R | S | S | S | S | S | R | S | R | S | S | S | R | R | R | S | R |
| Erythromycin | R | S | S | S | R | S | S | R | R | R | R | R | S | S | R | R | R | S | S | S | R | S | R | R | S |
| Linezolid | S | S | S | S | S | S | S | S | S | S | S | S | S | S | S | S | S | S | S | S | S | S | S | S | S |
| Clindamycin | R | S | S | S | R | S | S | R | R | R | R | R | S | S | R | R | R | S | S | S | R | S | R | R | S |
| Gentamycin | S | R | S | R | R | S | R | R | R | S | S | S | R | R | S | R | R | S | S | R | R | R | R | S | S |
| Qxacillin | R | R | R | R | R | R | R | R | R | R | R | R | R | R | R | R | R | R | R | R | R | R | R | R | R |
| Levofloxacin | S | R | S | S | R | S | S | R | R | S | S | S | S | S | R | S | R | S | S | S | R | R | R | S | R |
| Tetracycline | S | R | R | S | R | S | R | R | R | S | S | S | R | R | S | R | R | R | R | R | R | R | R | S | S |
| Tigecycline | S | S | S | S | S | S | S | S | S | S | S | S | S | S | S | S | S | S | S | S | S | S | S | S | S |
| Rifampicin | S | I | S | S | I | S | S | R | R | S | S | S | S | S | S | R | S | S | S | S | R | I | S | S | S |
| Teicoplanin | S | S | S | S | S | S | S | S | S | S | S | S | S | S | S | S | S | S | S | S | S | S | S | S | S |
| Trimethoprim/ Sulfamrthoxazole | R | S | S | S | S | S | S | S | S | S | R | R | S | S | R | S | S | R | R | S | S | S | R | S | S |

*R: complete resistance, S: susceptible,I: Intermediated

Table S2: The efficiency of plating of phage vB_SauP_ASUmrsa123.

| No. of MRSA strain | EOP | EOP after calculation | PFU / ml |
| --- | --- | --- | --- |
| ST1 ATCC OQ564500.1(Host) | 1.0 | 1.0 | 10^9^ |
| ST 2 | - | - | - |
| ST 3 | ( 7 × 10^-1^) | 0.7 | 10^8^ |
| ST 4 | (1.3× 10^-4^) | 0.00013 | 10^4^ |
| ST 5 | (7× 10^-7^) | 0.0000007 | 10^2^ |
| ST 6 | (3.0× 10^-1^) | 0.3 | 10^8^ |
| ST 7 | (7.5 ×10^-2^) | 0.075 | 10^6^ |
| ST 8 | - | - | - |
| ST 9 | - | - | - |
| ST 10 | (3.8 ×10^-6^) | 0.0000038 | 10^3^ |
| ST 11 | (6× 10^-1^) | 0.6 | 10^8^ |
| ST 12 | 1.0 | 1.0 | 10^9^ |
| ST 13 | (2.5×10^-2^ ) | 0.025 | 10^7^ |
| ST 14 | (7.5×10^-2^ ) | 0.075 | 10^7^ |
| ST 15 | (3.0×10^-2^ ) | 0.03 | 10^7^ |
| ST 16 | - | - | - |
| ST 17 | - | - | - |
| ST 18 | 1.0 | 1.0 | 10^9^ |
| ST 19 | (6.3 ×10^-1^) | 0.63 | 10^8^ |
| ST 20 | (5×10^-2^ ) | 0.05 | 10^7^ |
| ST 21 | - | - | - |
| ST 22 | - | - | - |
| ST 23 | - | - | - |
| ST 24 | - | - | - |
| ST 25 | - | - | - |


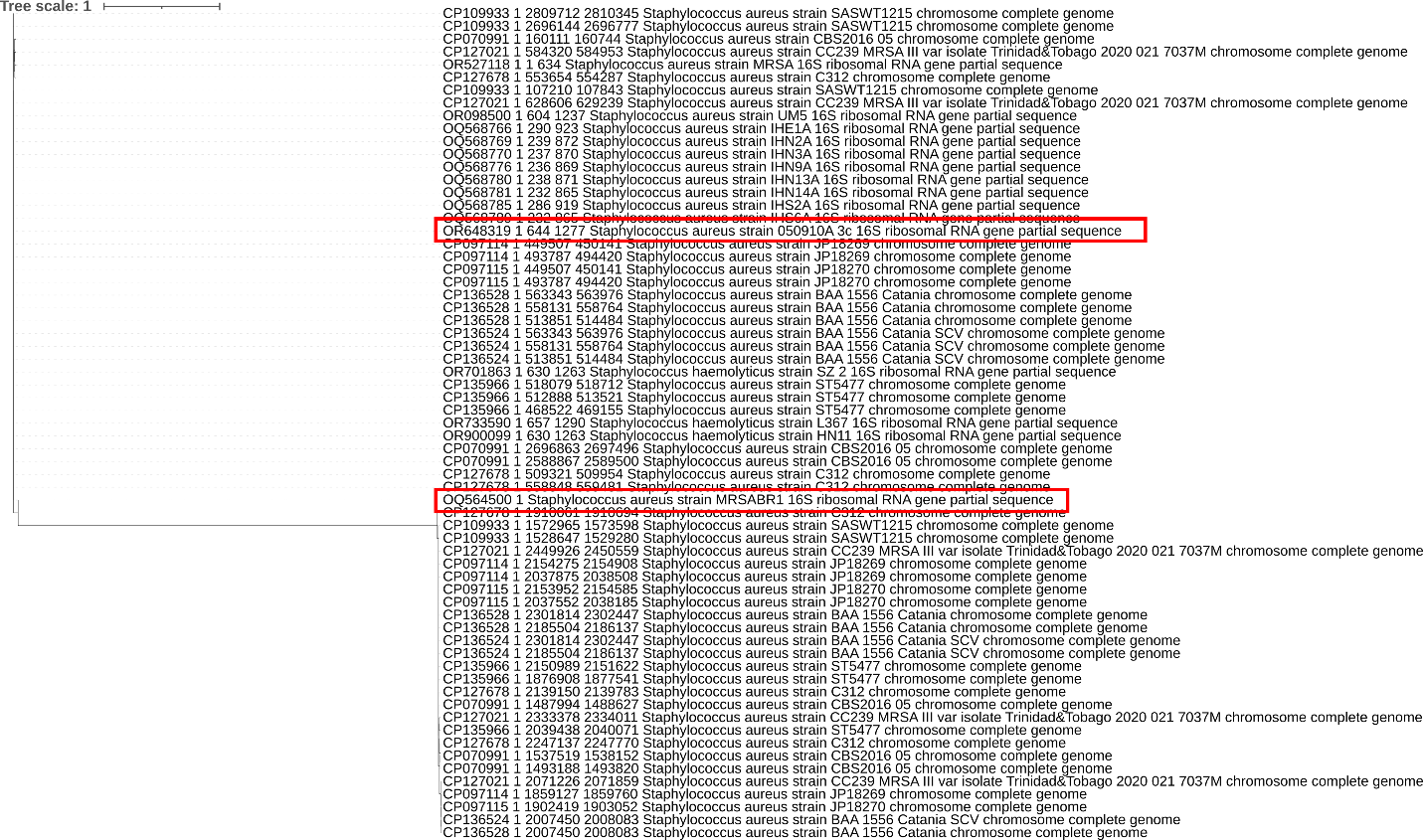


**Figure S1**: 16S rRNA Phylogenetic relationship of the two *S. aureus* MRSA host bacteria OQ564500 and OR527118 with other *S. aureus* isolates in GenBank database.

**Table S3**: Phage vB_SauP_ASUmrsa123 genes predicted functions.

| Type | Location | Start | Stop | Strand | Function |
| --- | --- | --- | --- | --- | --- |
| CDS | Staphylococcus_165+90 | 165 | 254 | + | hypothetical protein |
| CDS | Staphylococcus_488-210 | 488 | 279 | - | hypothetical protein |
| CDS | Staphylococcus_575+132 | 575 | 706 | + | inner core protein |
| CDS | Staphylococcus_719+174 | 719 | 892 | + | arstotzka protein |
| CDS | Staphylococcus_972+126 | 972 | 2136 | + | major head protein |
| CDS | Staphylococcus_2152+975 | 2152 | 3126 | + | upper collar protein |
| CDS | Staphylococcus_3245+279 | 3245 | 3884 | + | Phage lower collar protein |
| CDS | Staphylococcus_3898+852 | 3898 | 5733 | + | Putative major teichoic acid biosynthesis protein C |
| CDS | Staphylococcus_5852+279 | 5852 | 6525 | + | Phage lysin, N-acetylmuramoyl-L-alanine amidase (EC 3.5.1.28) |
| CDS | Staphylococcus_6660+225 | 6660 | 7293 | + | Phage tail fiber |
| CDS | Staphylococcus_7466+126 | 7466 | 7591 | + | Phage minor tail protein |
| CDS | Staphylococcus_7649+105 | 7719 | 8899 | + | Phage tail fiber |
| CDS | Staphylococcus_9422+420 | 9422 | 9841 | + | Phage holin |
| CDS | Staphylococcus_9819+231 | 9819 | 11259 | + | CHAP domain-containing protein |
| CDS | Staphylococcus_11919-552 | 13718 | 11680 | - | DNA polymerase (EC 2.7.7.7), phage-associated |
| CDS | Staphylococcus_13811-138 | 14476 | 13586 | - | terminase |
| CDS | Staphylococcus_14988-90 | 14988 | 14899 | - | hypothetical protein |
| CDS | Staphylococcus_15363-258 | 15363 | 15106 | - | hypothetical protein |
| CDS | Staphylococcus_15345+132 | 15345 | 15476 | + | hypothetical protein |
| CDS | Staphylococcus_15914-189 | 15914 | 15726 | - | hypothetical protein |
| CDS | Staphylococcus_16192-177 | 16192 | 16016 | - | hypothetical protein |
| CDS | Staphylococcus_16611-270 | 16611 | 16342 | - | Single stranded DNA-binding protein, phage-associated |
| CDS | Staphylococcus_16895-111 | 16895 | 16785 | - | hypothetical protein |
| CDS | Staphylococcus_17154-255 | 17154 | 16900 | - | hypothetical protein |
